# The identification of risk factors associated with patient and healthcare system delays in the treatment of tuberculosis in Tabriz, Iran

**DOI:** 10.1186/s12889-018-5066-9

**Published:** 2018-01-24

**Authors:** Mohammad Ebrahimi Kalan, Hassan Yekrang Sis, Vinaya Kelkar, Scott H. Harrison, Gregory D. Goins, Mohammad Asghari Jafarabadi, Jian Han

**Affiliations:** 1 Department of Biology, North Carolina Agricultural and Technical State University, Greensboro, NC 27411 USA; 2 Tuberculosis and Lung Diseases Research Center, Tabriz University of Medical Sciences and Health Services, Tabriz, East Azerbaijan Iran; 3 Road Traffic Injury Research Center, Tabriz University of Medical Sciences and Health Services, Tabriz, East Azerbaijan Iran; 4 Department of Statistics and Epidemiology, Faculty of Health, Tabriz University of Medical Sciences and Health Services, Tabriz, East Azerbaijan Iran

**Keywords:** Tuberculosis, Patient delay, Healthcare system delay, Delay, Tabriz, Iran

## Abstract

**Background:**

Tuberculosis (TB) is a serious health concern, particularly in developing countries. Various delays, such as patient delay (PD) and healthcare system delay (HSD) in the TB process, are exacerbating the disease burden and increasing the rates of transmission and mortality in various global communities. Therefore, the aim of this study is to identify risk factors associated with PD and HSD in TB patients in Tabriz, Iran.

**Methods:**

A cross-sectional study was conducted on 173 TB patients in Tabriz, Iran from 2012 to 2014. Patients were interviewed with a semi-structured questionnaire. Frequencies and percentages were reported for patient categories of sex, age, and education. The median and interquartile range (IQR) were reported for the time intervals of delays. Univariate and multivariate logistic regressions of delay in respect to socio-demographic and clinical variables were performed. Statistical significance was set at *p* < 0.05.

**Results:**

The median values for delays were 53 days for HSD (IQR = 73) and 13 days for PD (IQR = 57). Odds ratios (OR) associated with PD were: employed vs. unemployed (OR = 5.86, 95% CI: 1.59 to 21.64); public hospitals vs. private hospitals (OR = 2.64, 95% CI: 1.01 to 6.85); ≥ 3 vs. < 3 visits to health facilities before correct diagnosis (OR = 2.35, 95% CI: 1.08 to 5.11); and male vs. female (OR = 2.28, 95% CI: 1.29 to 4.39). The OR associated with HSD were: ≥ 3 vs. < 3 visits to health facilities before correct diagnosis (OR = 9.44, 95% CI: 4.50 to 19.82), without vs. with access to TB diagnostic services (OR = 3.56, 95% CI: 1.85 to 6.83), and misdiagnosis as cold or viral infection vs. not (OR = 2.62, 95% CI: 1.40 to 4.91).

**Conclusions:**

The results provide for an important understanding of the risk factors associated with PD and HSD. One of the major recommendations is to provide more TB diagnostic knowledge and tools to primary health providers and correct diagnoses for patients during their initial visit to the health care facilities. The knowledge generated from this study will be helpful for prioritizing and developing strategies for minimizing delays, initiating early treatment to TB patients, and improving TB-related training programs and healthcare systems in Tabriz, Iran.

## Background

Tuberculosis (TB) is a global public health problem that continues to remain a serious health concern, particularly for developing countries [1–3], and is being confronted with aggressive global strategies for monitoring and control [4, 5]. According to the TB global report in 2015, there were an estimated 10.4 million incident cases and 1.8 million deaths from TB [2]. More than 95% of TB deaths occur in low- and middle-income countries [6]. For more than a decade, the World Health Organization (WHO) has introduced various programs to improve the quality of directly observed treatment, short-course strategy (DOTS) and reducing the burden of TB globally [2, 4, 6, 7]. Although efforts for treatment and diagnostic approaches for active TB have been aggressively pursued [8, 9], health care systems are still having conspicuous problems with timely diagnosis and proper treatment [1, 10]. Without proper treatment, a TB patient, on average, can infect 15–20 people annually [4]. Therefore, early diagnosis and effective treatment are fundamental elements that need to be addressed for all national TB programs (NTP) [1].

A primary goal of every NTP is to stop the transmission of TB within the community, by minimizing the time between onset of symptoms and DOTS initiation [11, 12]. Previous studies have shown that the factors of patient delay (PD) and healthcare system delay (HSD) in TB diagnosis and treatment increase the probability of TB transmission in the community as well as the mortality rate [10, 13, 14]. The issue of HSD of TB is a crucial challenge in both developed and developing countries [15], and HSD may be considered as a measure of the level of TB awareness and the efficiency of the country’s NTP [10].

Nine countries contributed a total of 95% of TB burden in Eastern Mediterranean region in 2010. These countries were Pakistan, Afghanistan, Sudan, Morocco, Somalia, Iraq, Egypt, Yemen, and the Islamic Republic of Iran [16]. In a region-based study by WHO in 2006 [17], the Islamic Republic of Iran contributed only 6% of the TB burden to the Eastern Mediterranean Region (EMRO) with a wide range of overall delay (OD) (5–728 days). In this region-based study, with a total sample size of 5053 patients, Iran contributed 800 TB patients, from which more than half of them (51%) reported a delay between the onset of symptoms and treatment [17]. Even though Iran is considered as an upper-middle-income country [18] with an advanced healthcare system [19], it borders on countries with high levels of TB prevalence including Iraq, Afghanistan, Pakistan, and newly independent countries from the former Soviet Union. These border countries present a particular challenge for the Iranian NTP [20, 21]. Therefore, it is imperative to reduce TB treatment delays in Iran so as to improve the Iranian NTP, and to mitigate transmissions into Iran from external sources.

Current literature does not provide an accepted “time interval” definition of delays for TB patients [22, 23]. Evaluations and reasons for delays can be as diverse as the various local health facilities and regional epidemiological aspects of TB [24]. One study reported that the main delay of TB diagnosis and treatment in Iran was the time taken by medical doctors to diagnose TB in symptomatic patients [25]. Moreover, a study in northern Iran demonstrated that the knowledge about diagnosis, treatment, and monitoring of TB in medical students was poor [26]. Another study showed that only 57% of all TB patients in Iran are diagnosed correctly when first attending a healthcare facility [20], which is far below the WHO target objective of 70% case detection of TB from 1997 onwards [27]. The WHO target objective is essential for all NTPs, worldwide, as relates to an overall goal for eliminating TB from the human population [6].

There is much variability across studies regarding the time interval of delays in TB patients [12, 28–32]. Two systematic reviews conducted across sets of multiple studies have reported medians of PD and HSD to range from 6 to 267.7 days and 2-120 days, respectively [23, 33]. Various factors have been reported as predictors for PD and HSD including lower education (PD) [34], being female (PD), being male (HSD), smoking (PD), older age (PD), initial visit to the private sector (HSD), cough (PD) [10, 11, 14, 27, 35, 36], history of contact with TB patient (HSD) [37], financial problem (HSD), consultation at a public hospital before diagnosis (HSD) [38], and visiting healthcare facilities more than once (PD) [1] or twice (HSD) [36, 39].

To the best of our knowledge, the potential risk factors associated with PD and HSD have not been extensively researched in the northwest of the Iran which includes Tabriz, a city with a population of around 1.5 million [40]. Tabriz has only one dedicated Tuberculosis and Lung Diseases Research Center (TLDRC). In Iran, typical procedures for TB diagnosis are as follows: general physicians from private and public hospitals/clinics can diagnose TB based on patients’ symptoms; however, all TB cases need to be referred to a TLDRC for further laboratory tests and final confirmation of diagnosis. Furthermore, DOTS treatment to patients is given at TLDRC. Even though this region of Iran is not considered to have a high burden of TB, it is important to note that, in the regions where TB cases are not common, it is generally difficult to attain a high degree of immediate treatment action by healthcare facilities [10]. Therefore, identifying the source of delay in Tabriz and the surrounding northwest region of Iran is crucial for early diagnosis and proper treatment of TB locally [2]. The aim of this study was to identify the time intervals of delays and to predict associated risk factors for TB in Tabriz, Iran. In this study, the OD is split into PD and HSD, so as to allow a better identification of risk factors associated with these two types of delays [41].

## Methods

### Study design, setting, and participants

This cross-sectional study was conducted in Tabriz, the capital city of East Azerbaijan province at the northwest of Iran. All eligible diagnosed pulmonary TB patients who had medical records at the TLDRC in Tabriz from 2012 to 2014 participated in the study. Pulmonary TB cases were defined based on both WHO and Iranian national TB guidelines [9, 12, 42]. Bacteriological confirmation was not used as an inclusion criterion for the present study, but all patients had to receive a TB DOTS for 6 months from a TLDRC. This study was approved by the Students Research Committee (No. 5/46/210) and Ethical Regional Committee (No. 5/4/9632) of Tabriz University of Medical Sciences and Health Services, Tabriz, Iran. The Institutional Review Board at the North Carolina Agricultural and Technical State University (NCAT) received the approved letters.

Patients were aware of the objectives, steps, and expected outcomes of the research. All patients who participated in this study signed the written informed consent. Overall, 185 TB patients were admitted to the TLDRC from January 2012 to December 2014. Only patients aged over 18 years old were recruited for the study. TB patients with foreign nationality, previous history of TB therapy equal to or more than twice, and prisoners who were imprisoned during the study period were excluded from this study. After exclusion, a total of 173 TB patients were included for further data analysis in this study.

### Data collection

Data were collected using the standardized questionnaire from WHO’s EMRO study [1], which was translated into Persian for members of the Iranian population. All TB patients were registered in the NTP system (NTP) when they first attended TLDRC in Tabriz, Iran. Clinical and socio-demographic information was obtained from the NTP after receiving the verbal informed consent from patients through the phone. Information associated with risk factors of PD and HSD in TB patients was collected through the interview process after receiving written informed consent from patients. The interviews were performed at each patient’s home. Patients were informed about the objectives and significance of the study before the interview.

### Definitions

#### Directly observed treatment, short-course (DOTS)

**Fig. 1:**
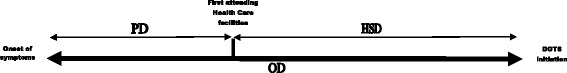
Conceptual framework of delays and their definitions (modified from a WHO region-based study) [17]. PD: patient delay; HSD: health care system delay; and OD: overall delay. DOTS: directly observed treatment, short-course

This is a strategy used to reduce the incidence and spread of tuberculosis (TB) cases. This strategy has two goals, to ensure that the TB patient completes the anti-TB regimen, to best promote healing and cure; and to prevent drug resistance that can otherwise occur from improper and incomplete use of anti-TB drugs [43]. The time interval from the inception of symptoms until the initiation of DOTS strategy is defined as overall delay (OD), which is the sum of two delays, PD and HSD (Fig. 1).

#### Patient delay (PD)

The time interval (in days) from the onset of symptoms until first attending healthcare facilities.

#### Healthcare system delay (HSD)

The time interval (in days) between the date of initial presentation at a healthcare facility and the initiation of the DOTS regimen.

#### Primary health care (PHC)

This level of care involves family physicians (general practitioners) who provide systematic referral to other levels of care such as secondary health care (SHC) or tertiary health care (THC) for situations needing more specialized diagnosis and treatment.

#### Secondary health care (SHC)

This level of care is a specialized unit within the healthcare system. Health workers provide outpatient or inpatient treatment as well as rehabilitation services for those referred from primary health care practitioners.

#### Tertiary health care (THC)

This level of care involves specialized consultative outpatient or inpatient treatment that is at an especially advanced level for specialized surgeries, rehabilitation services, and provisioning of medicines, other medical supplies and laboratory services.

### Statistical analysis

The analysis was performed in five steps. First, frequency and percentage were calculated for categorical variables such as age, gender, education and occupation, while medians and inter-quartile ranges (IQR) were calculated for continuous variables with a non-normal distribution. Consistent with previous studies, the cutoff points for delays were defined as the median value obtained for each delay [1, 10, 14, 44]. Less than or equal to median was considered as “without delay” and greater than median was considered as “with delay”. Second, the new and retreated TB patients (with only one course of previous treatment) were not separated in the current study, because there was no significant difference between them tested by Kaplan-Meier survival analysis. Third, to test the association between delays (presence: delays >median, absence: delays ≤ median) with categorical variables, the Pearson’s chi-square or the Fisher’s exact test was applied at *α* = 0.05 level. Association between delays (absence, presence) with the type of healthcare system (private or public) were made using the Mood’s median test at *α* = 0.05 level. Next, to investigate the potential risk factors associated with delays, univariate and multivariate analyses were applied. The one-way analysis of variance (ANOVA) was used to determine the impact of various level of the health care facilities on the diagnosis of TB. Finally, to evaluate adjusted odds ratios and corresponding 95% Confidence Intervals (95% CI) for risk factors of HSD and PD, the multivariate logistic regression was applied. Factors associated with delays were analyzed using multivariate regression analysis [14, 22, 45]. Data analysis was conducted using the Statistical Packages for the Social Sciences (SPSS) version 22 (Released 2013. IBM SPSS Statistics for Windows, Version 22.0. Armonk, NY: IBM Corp). The level of significance was considered at *α =* 0.05.

## Results

### Socio-demographic and clinical characteristics of the participants

The mean age of the patients was 55.6 years old (median 59, range 18-90). Among all participants, 88 (50.9%) were male and 85 (49.1%) were female. Among all participants, 79 (45.7%) were illiterate, 84 (48.6%) unemployed, and 106 (61.3%) married. As shown in Table 1, the median (IQR) of PD and HSD were 13 (57) and 53 (73), respectively.

**Table 1:** Descriptive statistics of PD and HSD (days) in TB patients. Tabriz, Iran 2012-2014

Type of delay^a^	PD	HSD
Mean (SD^b^)	46.2 (82.5)	75.3 (89.2)
Median (IQR^c^)	13 (57)	53 (73)
Range	0-453	1-726

^a^All delays are based on days. ^b^Standard Deviation. ^c^Interquartile range

About 93 (58.5%) of the patients attended clinics or hospitals three or more times between the onset of symptoms and the time at which they were diagnosed with TB. The major symptoms that motivated patients for their first-time visit to the health care facilities were coughing, for 170 (98.3%) of the patients, and unusual sputum, for 161 (93.1%) of the patients. About 102 (59%) of the patients were misdiagnosed as having a cold or viral infection, 13 (7.5%) were misdiagnosed as having asthma, and 10 (5.8%) were misdiagnosed as having chronic obstructive pulmonary disease (COPD).

There was a significant association between misdiagnosis as cold or viral infection and the different level of healthcare facility (Table 2). Misdiagnoses as cold or viral infection in TB patients, upon their first-time visit to a healthcare facility, varied significantly based upon type of facility: PHC (58.8%), SHC (36%), and THC (4.6%) (*p* < 0.001). Misdiagnosis as asthma was also statistically significant at these different varieties of facilities (*p* < 0.003). The PHC facilities contributed to 50% of the first-time correct diagnoses of TB that were referred to TLDRC, and the SHC and THC facilities contributed to 34.6% and 15.4% of first-time correct diagnoses, respectively. In 173 of the patients studied, only 26 (15%) of the patients were correctly diagnosed as TB in their first visit to health facilities.

**Table 2:** Diagnosis before TB confirmation by the level of health care facilities in TB patients, Tabriz, Iran 2012-2014

First diagnosis	PHC^a^ N (%)	SHC^b^ N (%)	THC^c^ N (%)	*P*-value ^e^
TB (*N* = 26)	13 (50)	9 (34.6)	4 (15.4)	0.658
Cold or viral infection (*N* = 102)	60 (58.8)	37 (36.3)	5 (4.6)	< 0.001
Asthma (*N* = 13)	4 (30.8)	3 (23.1)	6 (46.2)	0.003
COPD^d^ (N = 10)	4 (40)	6 (60)	0 (0.0)	0.199
Others (*N* = 21)	6 (28.6)	12 (57.1)	3 (14.3)	0.139

^a^Primary Health Care, ^b^Secondary Health Care, ^c^Tertiary Health Care, ^d^COPD: Chronic Obstructive Pulmonary Disease. ^e^*P*-values are based on Analysis of Variance (ANOVA) test

### Factors associated with patient delay (PD)

**Fig. 2:**
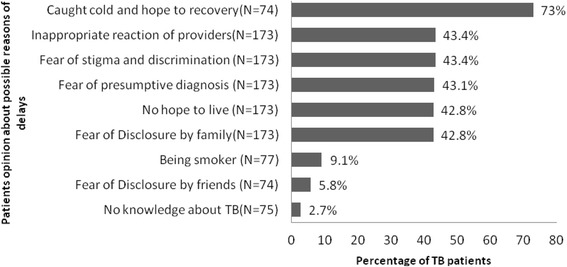
Patients’ opinions on possible reasons of delay in diagnosis and treatment of TB in Tabriz, Iran, 2012-2014

Patients’ opinions on possible personal reasons for delay in visiting health facility for receiving diagnosis and treatment are shown in Fig. 2. There were 43.4% of the patients who thought that the inappropriate reaction of health services provider was one of the reasons for delayed diagnosis and treatment of TB; 43.1% were scared about presumptive diagnosis; and 73% mentioned that they thought their disease was a regular cold and hoped to recover.

Clinical and demographical factors associated with PD are described in Table 3. PD was significantly more frequent among male compared to female patients. (61.4% vs. 38.6%; *p* < 0.005). The significant risk factors for PD were being employed (5.86-fold increased risk), attending public hospitals or clinics for the first time after the onset of TB symptoms (2.64-fold increased risk), and being male (2.28-fold increased risk). Age, marital status, educational level, history of TB contact, economical affordability of diagnostic services, and misdiagnosis were not strongly associated with the risk of PD. Table 4 indicated that 66.2% of patients attending public hospitals or clinics had PD compared to the 35% of patients attending private hospitals or clinics (*p* < 0.05).

**Table 3:** The risk factors associated with patient and health care system delay in TB patients in Tabriz, Iran 2012-2014 (*N* = 173)

	Number (%)	Patient delay^a^		Health care system delay^b^	
Factors		N (%)^c^	Crude OR (95% CI)	N (%)^c^	Crude OR (95% CI)
Gender					
Female^†^	85 (49.1)	33 (38.8)	1	48 (56.5)	1
Male	88 (50.9)	53 (60.2)	2.28 (1.29 to 4.39)*	37 (42.0)	0.55 (0.30 to 1.022)
Age group					
≤ 30^†^	41 (23.7)	16 (39)	1	21 (51.2)	1
> 30	132 (76.3)	70 (53)	1.76 (0.86 to 3.60)	64 (48.5)	1.11 (0.55 to 2.24)
Education					
Illiterate/W&R^d †^	79 (45.7)	40 (50.6)	1	41 (51.9)	1
Primary-Secondary	55 (31.8)	29 (52.7)	1.08 (0.54 to 2.16)	23 (41.8)	0.66 (0.33 to 1.33)
University/Higher	39 (22.5)	17 (43.6)	0.75 (0.348 to 1.63)	21 (53.8)	1.08 (0.501 to 2.333)
Occupation					
Unemployed^†^	84 (48.6)	40 (47.8)	1	47 (56.0)	1
Employed	19 (11.0)	16 (84.2)	5.86 (1.59 to 21.64) *	8 (42.1)	0.57 (0.20 to 1.56)
Student	14 (8.1)	2 (14.3)	0.18 (0.03 to 0.87) *	11 (78.6)	2.88 (0.75 to 11.10)
Retired	36 (20.8)	18 (15.0)	1.10 (0.50 to 2.40)	12 (33.3)	0.39 (0.17 to 0.89) *
Farmer	20 (11.6)	10 (50.0)	1.10 (0.415 to 2.91)	7 (35.0)	0.42 (0.154 to 1.16)
Marital status					
Married^†^	106 (61.2)	52 (49.5)	1	47 (44.3)	1
Single	29 (16.8)	14 (48.3)	0.96 (0.42 to 2.20)	15 (51.7)	1.34 (0.59 to 3.06)
Divorced	6 (3.5)	5 (83.3)	5.19 (0.58 to 45.95)	3 (50.0)	1.25 (0.24 to 6.50)
Widow/death	32 (18.5)	15 (46.9)	0.91 (0.41 to 2.02)	20 (62.5)	2.09 (0.92 to 4.71)
Smoking					
Never^†^	70 (40.5)	3 (45.7)	1	34 (48.6)	1
Current	75 (43.4)	39 (52.0)	1.28 (0.67 to 2.47)	38 (50.7)	1.08 (0.56 to 2.08)
Ex-smoker	28 (16.2)	15 (53.6)	1.37 (0.56 to 3.30)	13 (46.4)	0.91 (0.38 to 2.208)
Household size					
< =3 people^†^	67 (38.7)	38 (56.7)	1	30 (44.8)	1
> 3 people	106 (61.3)	48 (45.3)	1.58 (0.85 to 2.93)	55 (51.9)	0.75 (0.40 to 1.38)
History of TB contact					
Yes^†^	35 (20.2)	13 (37.1)	1	13 (37.1)	1
No	138 (79.8)	73 (52.1)	1.901 (0.88 to 4.07)	72 (52.2)	1.84 (0.86 to 3.95)
First misdiagnosis					
Cold or viral infection					
No^†^	71 (41.0)	39 (54.9)	1	26 (35.2)	1
Yes	102 (59.0)	47 (46.1)	1.42 (0.77 to 2.62)	60 (58.8)	2.61 (1.40 to 4.91)*
Asthma					
No^†^	160 (92.5)	80 (50.0)	1	77 (48.1)	1
Yes	13 (7.5)	6 (46.2)	1.16 (0.37 to 3.62)	8 (51.5)	0.58 (0.18 to 1.84)
COPD					
No^†^	163 (94.2)	82 (50.3)	1	80 (49.1)	1
Yes	10 (5.8)	4 (40.0)	1.51 (0.41 to 5.58)	5 (50.0)	0.96 (0.26 to 3.45)
The number of attending health care facilities					
< 3^†^	65 (41.4)	36 (55.4)	1	15 (18.1)	1
≥ 3	93 (58.6)	37 (50.7)	0.54 (0.28 to 1.03)	68 (81.9)	9.44 (4.50 to 19.82)**
Center of first contact					
Traditional healer^†^	6 (3.5)	3 (50)	1	4 (66.7)	1
Public	77 (44.5)	51 (66.2)	2.64 (1.01 to 6.85)*	36 (46.8)	0.43 (0.7 to 2.5)
Private	90 (52.0)	32 (35.6)	0.55 (0.10 to 2.89)	45 (50.0)	0.50 (0.08 to 2.8)
Level of health care facilities					
PHC^e†^	83 (48.0)	44 (53.0)	1	43 (51.8)	1
SHC^f^	71 (41.0)	34 (47.9)	0.81 (0.43 to 1.53)	31 (43.7)	0.72 (0.38 to 1.36)
THC^g^	19 (11.0)	8 (42.1)	0.64 (0.23 to 1.76)	11 (57.9)	1.27 (0.46 to 3.50)
Financial affordability for diagnostic tests					
Yes^†^	66 (38.2)	39 (59.1)	1	20 (30.3)	1
No	107 (61.8)	47 (43.1)	0.54 (0.29 to 1.01)	65 (60.7)	3.56 (1.85 to 6.83)**

^†^Reference category, ^a^Patient delay > 13 days, ^b^Health care system delay> 52 days, ^c^All percentages are reported within dependent variables (associated risk factors), ^d^Writing and reading, *p < 0.05, ** *p* < 0.001, ^e^Primary Health Care, ^f^Secondary Health Care, ^g^Tertiary Health care

P-values are based on binomial logistic regression. *OR* indicates odds ratio, *CI* confidence interval

**Table 4:** Difference observed in various delays between private and governmental clinics/hospitals in TB patients in Tabriz, Iran 2012-2014

Type of Delay	Public Hospital/Clinics N = 77		Private Hospital/Clinics N = 90	*P*-value^†^
Patient delay	Yes N (%)^a^	51 (66.2)	32 (35.0)	0.001
	Range (days)	0-365	0-453	
Health care system delay	Yes N (%)^b^	36 (46.7)	45 (50)	0.62
	Range (days)	1-425	1-726	

^a^Median PD > 13 days, ^b^median HSD >53 days, ^†^*P*-value is based on Mood’s median test

### Factors associated with health care system delay (HSD)

Of 173 TB patients, 85 (49.5%) had HSD. Factors associated with HSD by clinical and demographical characteristics are described in Table 3. Attending health care facilities ≥ 3 times before TB diagnosis (9.44-fold increased risk), misdiagnosis as cold or viral infection (2.61-fold increased risk), and financial unaffordability for receiving diagnostic services (3.56-fold increased risk) were significant risk factors for HSD. The HSD in those TB patients that first attended private hospitals or clinics was more than the HSD in patients that first attended public hospital or clinic after the onset of symptoms (52.9% vs. 42.4%); however, the difference was not statistically significant. Being retired was a protective factor for HSD. There was no statistically significant relationship between the type of occupation as a risk factor and HSD. Age, marital status, being a smoker, educational level, and the history of TB contact did not appear to be related to HSD.

## Discussion

The present study identified the potential risk factors associated with PD and HSD for TB patients in the Tabriz, Iran. The major findings of the study are: (a) statistically significant risk factors for PD include: visiting public hospital/clinics (compared to private hospitals/clinics) after onset of symptoms; being employed (compared to being retired, unemployed, or being a homemaker), and being male; (b) statistically significant risk factors for increased HSD include: initial misdiagnosis as cold or viral infection, visiting any variety of health care facilities ≥ 3 times before diagnosis as TB, and financial unaffordability of diagnostic services; and (c) statistically significant risk factors for OD include: attending health care facilities ≥ 3 times before TB diagnosis, cold or viral infection, and financial unaffordability of the diagnostic services.

### Time interval and potential risk factors associated with PD

In this study, almost half of the patients (49.7%) had PD. The median time for the PD in this study was less than that in the national study of Iran (13 vs. 26 days) [17], possibly because of differing time periods of the studies (2006 vs. 2014) or no local regions, such as Tabriz, being separated in the national study. However, in a study done in Iran’s capital city (Tehran), the median PD was similar to the current study of Tabriz region [25]. Studies in other countries reported varying time intervals of the PD from 6 to 267.7 days [24, 33, 46–48]. In this study, the median of PD was almost similar to Egypt (12 days), Malaysia (14 days), and Ethiopia (15 days) [1, 25, 49–51], and lower than Nepal (50 days), Somalia (53 days), and China (93 days) [1, 52–54], and higher than Italy (7 days), Pakistan (9 days), and Taiwan (7 days) [1, 48, 55]. Therefore, PD is dependent on the country.

In agreement with previous studies, risk factors such as being a male, being employed, visiting health care facilities ≥3 times, and attending public hospitals (government hospitals) for the first time prior to diagnosis of TB were attributed to an increased PD [1, 15, 56–58]. Attending public hospitals was a risk factor for PD possibly because public hospitals are associated with prolonged waiting times, a high number of patients with diverse diseases, and lack of an adequate number of trained physicians. No significant association was observed between PD and financial unaffordability in receiving diagnostic tests. This could be explained by the fact that all TB treatment procedures in Iran are free, and are based on Iran’s NTP [42], from a patient first attending TB center until receiving adequate treatment by a physician. The risk for PD was not found to be related to a patient’s smoking status (current and ex-smoker), consistent with similar studies in Argentina and Mozambique [39, 59], and contrary to the findings of a study from the southern part of Iran [60]. In general, the PD in Iran is comparable to that in other developing and some developed countries [51]. However, since there is no defined time of delay in diagnosis and treatment of TB worldwide, this can be different based on population and the setting of study.

### Time interval and potential risk factors associated with HSD

The potential risk factors associated with HSD have never been explored for Tabriz, Iran. Even though this region is not considered as having a high rate of incidence for TB, we found that the median of HSD in this region of Iran is higher than national value (52 vs. 42 days) [1]. Based on previous studies, there is a wide range of median HSD values, from 2 to 120 days, in industrialized and developing countries [15, 17, 23, 61–63]. In this study, the median HSD was almost similar to studies in Malaysia (49 days), Vietnam (53 days), and New Zealand (49 days) [49, 62, 64]; lower than Mozambique (62 days), Pakistan (87 days), and Columbia (> 60 days) [1, 39, 52, 65]; and higher than Taiwan (29 days), India (28 days), Nigeria (7 days), and Angola (7 days) [63, 66–68]. Such a wide range of HSD among these countries demonstrates that HSD is still a big concern for TB treatment worldwide.

Only 15% of TB patients were diagnosed with TB when first attending a health care facility. The median number of visits to health care facilities prior to diagnosis of TB was four, which was almost similar to the report by Almeida et al. in 2015 [22]. While 93.1% of TB patients had reported the unusual sputum as their first symptoms prior to diagnosis of TB, the number of sputum smears requested by physicians was very low (14.5% of 66 TB patients). This is possibly because of a lack of awareness of TB symptoms among health care providers [69]. Our data also showed the 85% of the patients had a misdiagnosis when first attending health care facilities after the onset of TB-related symptoms. This is likely due to TB having similar symptoms to cold and other viral upper respiratory infections [70]. Current and previous studies show HSD is highly associated with more visits of health care facilities, and that this is likely also due to misdiagnosis at initial visit [36, 70]. The results of HSD highlight the importance of TB-related education for healthcare providers for addressing the challenges faced at TLDRC and other healthcare facilities in Tabriz, Iran.

Even though all the costs of diagnosis and treatment of TB are free in the TLDRC, only 15% of the patients attended TLDRC as their first action after the onset of TB symptoms. This is possibly because either the family physician referral system did not refer TB patients to the TLDRC due to misdiagnosis, lack of diagnostic awareness [71], or patients did not have any knowledge about the available services at the TB center. We believe that the long HSD before TB diagnosis and treatment in Tabriz needs to be reduced through improving quality and coverage of NTP [72]. Even though some patients reported different symptoms before TB diagnosis, none of them were significantly associated with PD or HSD. This could be related to either patients who seek care from informal health providers as an initial point of care or patients who self-medicate [73].

Although our study has elucidated, to some extent, the contributing potential factors associated with PD and HSD in Iran, some limitations need to be noted. In the current cross-sectional study, the sample is not representative of all TB patients in Iran; therefore, it is hard to generalize the results to the TB population Iran nationwide. Furthermore, the cross-sectional nature of our study does not allow us to deduce causality of the observed associations, and confounding variables could also mask the association. A larger nationwide sampling strategy should be pursued for future studies to explore the potential factors associated with PD and HSD in TB patients across Iran.

## Conclusion

The study has identified the potential risk factors associated with HSD, PD, and OD in Tabriz, Iran. The understanding of the potential risk factors related to these delays can minimize the length of time between the onset of symptoms and correct diagnosis, and will help to initiate treatment as soon as possible. Integrating the TLDRC with the public hospital, to minimize the attending time and increasing the rate of correct diagnosis after onset of symptoms, and improving training program for local public health services, particularly in the PHC system, will help to reduce these delays in the studied area.
